# Repetitive Neonatal Erythropoietin and Melatonin Combinatorial Treatment Provides Sustained Repair of Functional Deficits in a Rat Model of Cerebral Palsy

**DOI:** 10.3389/fneur.2018.00233

**Published:** 2018-04-13

**Authors:** Lauren L. Jantzie, Akosua Y. Oppong, Fatu S. Conteh, Tracylyn R. Yellowhair, Joshua Kim, Gabrielle Fink, Adam R. Wolin, Frances J. Northington, Shenandoah Robinson

**Affiliations:** ^1^Department of Pediatrics, University of New Mexico School of Medicine, University of New Mexico, Albuquerque, NM, United States; ^2^Department of Neurosciences, University of New Mexico School of Medicine, University of New Mexico, Albuquerque, NM, United States; ^3^Pediatric Neurosurgery, Johns Hopkins University, Baltimore, MD, United States; ^4^Neonatology, Department of Pediatrics, Johns Hopkins School of Medicine, Baltimore, MD, United States

**Keywords:** cerebral palsy, chorioamnionitis, hypoxia-ischemia, inflammation, social interaction, gait, touchscreen, cognition

## Abstract

Cerebral palsy (CP) is the leading cause of motor impairment for children worldwide and results from perinatal brain injury (PBI). To test novel therapeutics to mitigate deficits from PBI, we developed a rat model of extreme preterm birth (<28 weeks of gestation) that mimics dual intrauterine injury from placental underperfusion and chorioamnionitis. We hypothesized that a sustained postnatal treatment regimen that combines the endogenous neuroreparative agents erythropoietin (EPO) and melatonin (MLT) would mitigate molecular, sensorimotor, and cognitive abnormalities in adults rats following prenatal injury. On embryonic day 18 (E18), a laparotomy was performed in pregnant Sprague–Dawley rats. Uterine artery occlusion was performed for 60 min to induce placental insufficiency *via* transient systemic hypoxia-ischemia, followed by intra-amniotic injections of lipopolysaccharide, and laparotomy closure. On postnatal day 1 (P1), approximately equivalent to 30 weeks of gestation, injured rats were randomized to an extended EPO + MLT treatment regimen, or vehicle (sterile saline) from P1 to P10. Behavioral assays were performed along an extended developmental time course (*n* = 6–29). Open field testing shows injured rats exhibit hypermobility and disinhibition and that combined neonatal EPO + MLT treatment repairs disinhibition in injured rats, while EPO alone does not. Furthermore, EPO + MLT normalizes hindlimb deficits, including reduced paw area and paw pressure at peak stance, and elevated percent shared stance after prenatal injury. Injured rats had fewer social interactions than shams, and EPO + MLT normalized social drive. Touchscreen operant chamber testing of visual discrimination and reversal shows that EPO + MLT at least partially normalizes theses complex cognitive tasks. Together, these data indicate EPO + MLT can potentially repair multiple sensorimotor, cognitive, and behavioral realms following PBI, using highly translatable and sophisticated developmental testing platforms.

## Introduction

Cerebral palsy (CP) is the leading cause of motor impairment for children worldwide and typically results from perinatal brain injury (PBI) (1, 2). While preterm birth is a common etiologic antecedent, motor impairment and associated deficits can also arise from other insults to the developing central nervous system (CNS), including trauma and stroke. Notably, the scope of PBI has shifted over recent decades as more preterm infants survive (3–5), and the proportion of children with more severe motor impairment has increased in the USA (6). Within subpopulations of neonates with PBI, multiple injury mechanisms have been implicated, and emerging evidence strongly suggests that each newborn suffers a unique vulnerability to CNS injury from a combination of (1) inflammation from prenatal infection and/or hypoxia-ischemia (HI); (2) individualized risk from genetic and/or congenital predisposition and acquired prenatal exposures to drugs, toxins, and nutritional status; and (3) postnatal stresses, such as sepsis and surgery. Indeed, intrapartum events are implicated in the etiology of less than 12% of children with CP (7). Thus, there is an urgent need for safe, effective interventions for PBI, and subsequent CP and related deficits.

Infection and HI catalyze PBI by creating a toxic *in utero* and neural microenvironment that limits oxygen exchange and propagates inflammation during critical periods of neurodevelopment (8–15). Typically, infants with PBI present with injury to major white and gray matter structures that leads to reduced connectivity of developing cerebral networks. Subsequently, diverse functional deficits ensue with impairment in multiple motor, cognitive, and behavioral realms that precipitates poor educational progress during childhood (16–25). Chorioamnionitis (infection/inflammation of the amniotic fluid, membranes, and placenta) affects placental permeability and blood flow, facilitates HI and fetal transmission of inflammation, and is associated with a significant increase in systemic inflammation (26–29). Chorioamnionitis is common in both preterm and term infants (23, 24, 29–34). It affects approximately 40–80% of very preterm deliveries and 20–34% of deliveries at term (30, 33, 35). Chorioamnionitis is also recognized in as many as 42% of placentas from unremarkable pregnancies (29, 36). Notably, in term infants with HI encephalopathy, the presence of chorioamnionitis predicts decreased responsiveness to hypothermia treatment (8–10, 30–32, 37, 38) and magnesium sulfate (39). Because these current strategies for neonatal repair are less effective in the setting of chorioamnionitis, we sought to address this unmet need by testing promising neuroreparative agents using a preclinical model of chorioamnionitis.

Despite the wealth of epidemiological and clinical data related to chorioamnionitis and the development of motor deficits in children born preterm, little progress has been made in identifying interventions that mitigate the CNS injury that leads to CP. Indeed, ambulation, behavior, and cognition are complex tasks impacted by early CNS injury (40). To minimize deficits and optimize outcomes for children with CP, novel therapies are required to restore motor skills, sensation, behavior such as attention and social interaction, and cognition, including executive function. However, few novel therapies have directly addressed these complex and compound deficits, particularly the functional pillars of cognition and behavior with motor impairment. Here, we studied a combination therapy of the endogenous neuroreparative agents erythropoietin (EPO) and melatonin (MLT) in an established preclinical rat model that accurately encompasses the complete maternal-placental-fetal brain axis with intrauterine injury and recapitulates pathophysiology from extreme preterm birth. We chose a cocktail strategy to mitigate the multiple pathophysiological mechanisms that contribute to PBI, capitalize on innate CNS recovery, and respond to clinical recommendations on utility of single therapies (41–43). Furthermore, data from our labs and others, confirm combinatorial therapy with EPO + MLT may provide enhanced, synergistic neurorepair by (1) optimizing the genesis and survival of multiple neural cell lineages, including cells with high bioenergetic demands, such as oligodendrocytes, myelin sheaths, and ependyma with motile cilia, (2) normalizing excess calpain activity and its destruction of essential molecules during neurodevelopment, (3) reducing neuroinflammation and free radicals, and (4) limiting mitochondrial dysfunction and associated endoplasmic reticulum stress (44–55). Given this unique avenue for translation and targeted mechanisms of action, we tested the hypothesis that an extended postnatal EPO + MLT cocktail would mitigate gait, sensorimotor, cognitive, and behavioral changes associated with PBI, using highly translatable and sophisticated testing platforms that are similar to the ones used in humans, including digital gait analysis and touchscreen cognitive testing.

## Materials and Methods

The Institutional Care and Use Committee at the University of New Mexico Health Sciences Center, Boston Children’s Hospital and Johns Hopkins University approved all experimental procedures. For each experiment described, equal numbers of male and female pups were used, and data represents true *n* (individual pups) from at least two different dams per condition. Specifically, we adhered to accepted standards for rigorous study design and reporting to maximize the reproducibility and translational potential of our findings, as described by Landis et al. and in the ARRIVE guidelines (56–58). Animals of both sexes were randomized to experimental or sham control groups and EPO + MLT or vehicle treatments. All investigators were blinded to injury and treatment group during the conduct and analyses of each experiment. For each experiment, a power analysis was also performed to estimate the required sample size (G*Power 3.1.9.3). For these calculations, we used published and preliminary data to define the expected means and SDs for each group, and we exceeded the calculated number needed in every experiment. Separate cohorts of rats were used for open field, gait and social interaction, and touchscreen assessments.

### *In Utero* Injury: Chorioamnionitis

As placental structure and function is of significant clinical importance to neurologic sequelae in preterm survivors (59–61), we use a prenatal model of *in utero* transient systemic HI (TSHI) and intra-amniotic lipopolysaccharide (LPS) administration in pregnant rats (62–64). This approach capitalizes on an intact maternal–placental–fetal unit and is a model of PBI from extreme preterm birth (<28 weeks of gestation) that mimics dual intrauterine injury from placental underperfusion and chorioamnionitis (65). Briefly, under isoflurane anesthesia, a laparotomy is performed on embryonic day (E) 18. Uterine arteries are clamped for 60 min and followed by intra-amniotic injections of LPS (4 μg/sac; 0111:B4, Sigma, St. Louis, MO, USA) (62, 64, 65). Sham controls undergo anesthesia and laparotomy for 60 min without arterial clamping or LPS injections. Following closure of the laparotomy, dams receive narcotic pain medication, recover, and pups are born vaginally at E22, approximately equal to 30–32 weeks in human gestation. We have previously reported the effects of TSHI and LPS alone, and in concert, on CNS pathological hallmarks, functional motor outcomes, histologic placental injury, and expression of common pro-inflammatory cytokines (63, 64).

### EPO and MLT Combination Therapy

Erythropoietin and MLT are endogenous, developmentally regulated molecules that are individually most effective for neurorepair when administered in extended dosing regimens (45, 48, 66–69). Rodents are born at a time equivalent to the human third trimester, with P9 approximately equivalent to term in human gestation (70). Accordingly, we used an established, clinically relevant dosing regimen (47, 48, 55, 71), in which pups on postnatal day (P) 1 from all injured litters were individually randomized to receive either EPO (2,000 U/kg, R&D Systems, Minneapolis, MN, USA) plus MLT (20 mg/kg, Sigma), or vehicle (sterile saline). Subsequently, EPO was then administered intraperitoneally once daily from P1 to P5 and MLT was administered once daily from P1 to P10, comparable to dosing regimens used in human neonatal trials. When EPO was administered in isolation, it was given from P1 to P5 at 2,000 U/kg/dose as previously published (47, 48, 54, 55, 71, 72). Prior work has shown that shams do not exhibit any negative effects from EPO and MLT treatment (55), and thus to conserve resources, shams received only vehicle.

### Open Field

A circular open field arena (100 cm diameter) was placed in a quiet, well-lit room (130 lm), and was marked to divide the arena into three equally spaced, concentric circles labeled the center, neutral, and peripheral zones. At P28–P30, each rat was initially placed against the wall of the testing arena and allowed to explore for 15 min. Anymaze™ video-tracking software was used to record and measure open field behavior.

### Gait Analysis

Computerized gait analysis was performed on P25–P26 as previously described (62, 71). Briefly, digital video of each rat running on a backlit transparent treadmill set at 30 cm/s was acquired with a high-speed camera and analyzed using Digigait software (Mouse Specifics, Framingham, MA, USA). Digigait software analyses identifies individual paw prints and allows calculation of multiple gait metrics and kinematic measurements based on the position, area, and timing of each step. *In utero* chorioamnionitis induces a global injury. Thus, data from right and left hindlimbs were combined for analysis.

### Social Interaction

A standard paradigm was used to identify impaired social interaction in rats at P30–P32 (73–75). Briefly, 1 h prior to testing, each rat was isolated in a clean cage. For social interaction testing, two rats of the same sex and treatment group, but from different litters, were placed in a dimly lit (30 lm) circular testing arena (100 cm diameter) and recorded for 10 min using Anymaze™ video-tracking software. Each pair was counted as one social unit. Two observers blinded to the treatment group independently reviewed the trials and scored periods of social interaction (trailing, sniffing, grooming, playing, etc.). Intraclass coefficient was calculated for interrater reliability of social scoring. Olfactory testing for social and food odors confirmed primary sensory deficits were not related to the impaired social interaction observed in the injured animals.

### Touchscreen Testing

To better define deficits in cognitive realms, we use a touchscreen operant platform to test specific components of cognition and executive function commencing with mild food deprivation at P28, training at P35, initial testing at P42, and continuing through completion of the paradigms at approximately P90 (76–80). Briefly, using a separate cohort of rats, operant behavior was tested in a sound and light attenuating chamber (Med Associates, St. Albans, VT, USA). A pellet dispenser delivers 40 mg dustless pellets (Bioserv, Frenchtown, NJ, USA) into a magazine, and a houselight is located at one end of the chamber. The opposite end of the chamber houses a touch-sensitive screen (Conclusive Solutions, Sawbridgeworth, UK) overlaid by a black acrylic aperture plate, resulting in two separate touch areas for the rat to register a response. Stimulus presentation in the response windows and touches were controlled and recorded by KLimbic Software (Conclusive Solutions).

#### Pretraining

On P28, rats were first slowly reduced and then maintained at 85% free-feeding body weight. Rats were weighed and assessed for general health daily. The mild weight reduction was well tolerated. Prior to training, rats were acclimated to the 40 mg food pellet reward by provision of 25 pellets/rat in the home cage. Rats were then habituated to the operant chamber and to eating from the pellet magazine. Rats retrieving at least 48 pellets in 60 min were moved to a 4-stage training regimen. Rats first performed autoshaping, followed by three visual discrimination training sessions (76–79).

#### Discrimination and Reversal Learning

Following pretraining, all rats were tested on a pairwise discrimination-reversal paradigm during daily 60 min sessions. For discrimination learning, 2 novel, equiluminescent stimuli verified for rats, were presented in a spatially pseudo-randomized manner over 60-trial sessions (5-s inter-trial interval) (76–80). Responses at one stimulus yielded a reward, whereas responses at the other stimulus resulted in a 5 s time-out (singled by extinguishing the house light). Designation of initially reward stimulus was randomized across treatment. Stimuli remained on screen until a response was made. Rats were trained to an *a priori* criterion of greater than ≥80% correct responses for two consecutive days. Assessment of reversal learning began on the session after discrimination criterion was attained. For this test, the designation of stimuli as correct versus incorrect was reversed for each rat. Like discrimination, rats were tested on 60-trial daily sessions for reversal to an *a priori* criterion of ≥80% correct responses for two consecutive sessions. Errors on first presentation reversal trials were followed by correction trials which continued until a correct response was made, or the session ended. Failing criteria was set *a priori* at 21 sessions (days) for visual discrimination and 21 sessions (days) for reversal.

We recorded the following dependent measures during discrimination and reversal: total sessions, correct responses made, errors (incorrect responses made), correction errors (correction trials, reversal only), reaction time (time from stimulus presentation to touchscreen response), and magazine latency (time from touchscreen response to reward retrieval) (81). Discrimination performance was analyzed across all sessions required to reach criterion. To examine distinct phases of reversal (early perseverative and late learning) mediated by cortical and striatal subregions, respectively, we also analyzed errors and correction trials. Assuming a rat would achieve 50% correct by chance, perseveration was defined as sessions where performance was below 50% correct, and learning as performance from 50% correct to passing criterion, as previously described (81–83).

### Statistical Analysis

Statistical analyses were performed using SPSS25 (IBM, Armonk, NY, USA). For all analyses, data are represented as mean ± SEM, with *p* < 0.05 considered significant. For analysis of sham, vehicle-treated injury and EPO + MLT-treated injury groups, all parametric variables were tested for normal distribution with the Shapiro–Wilk test with Levene’s test to confirm homogeneity of variances. A two-way ANOVA was then performed with Bonferroni’s *post hoc* correction for multiple comparisons. For non-parametric variables such as passing criteria in touchscreen testing, a Kruskal–Wallis test with Dunn’s *post hoc* correction was performed.

## Results

### EPO + MLT Mitigates Disinhibition Following Prenatal Injury

**Figure 1:**
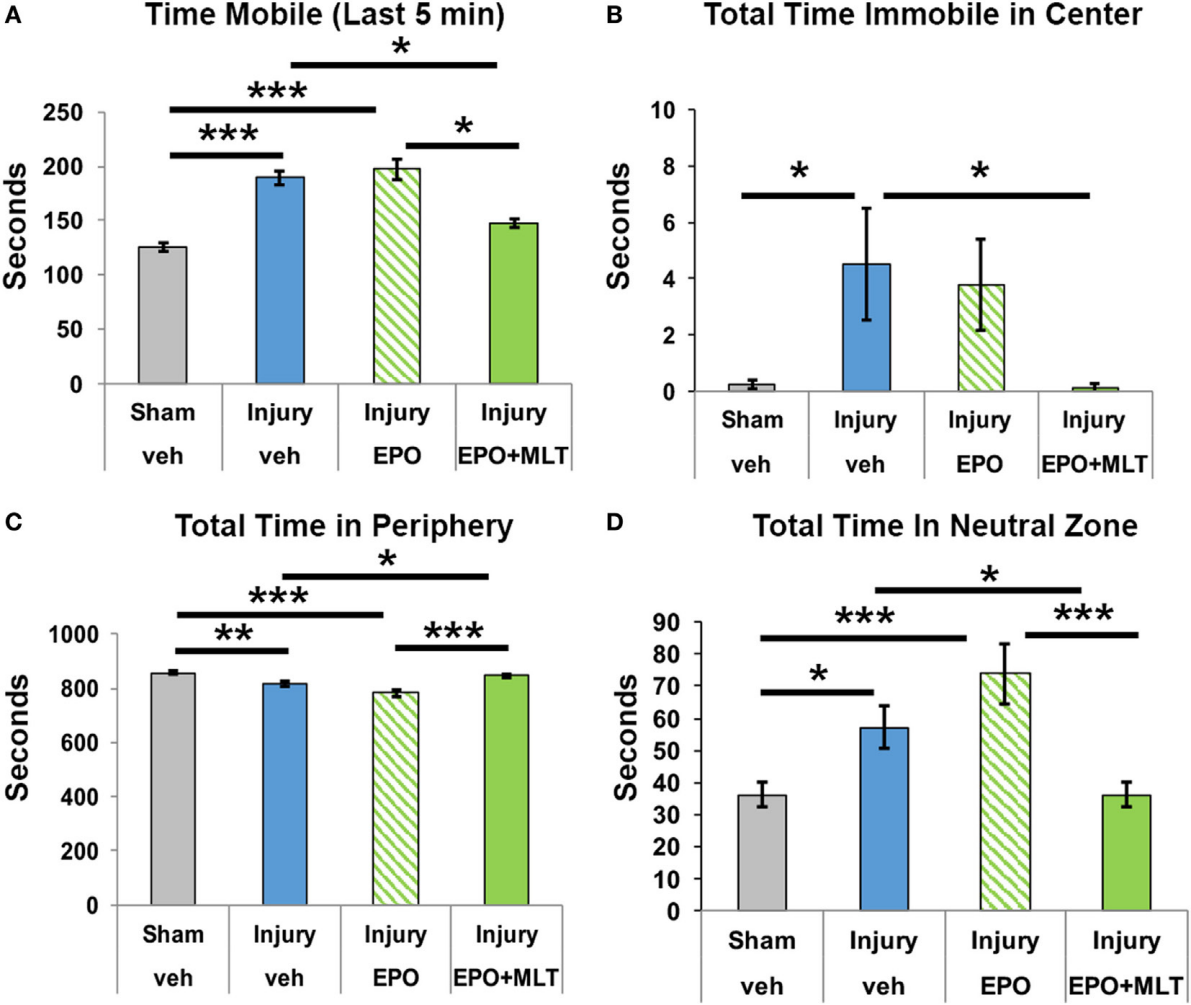
Open field testing of locomotion and disinhibition. **(A)** After prenatal injury, vehicle-treated rats are more mobile than shams, particularly in the last 5 min interval. Erythropoietin (EPO) + melatonin (MLT), but not EPO alone, normalizes the activity. **(B)** Rats typically avoid open areas. Vehicle-treated adult rats following prenatal injury spend more time immobile in the center zone than sham rats or EPO + MLT-treated rats. **(C)** Sham and EPO + MLT-treated injured rats spent more time in the peripheral zone than vehicle-treated or EPO-treated injured rats. **(D)** Similarly, vehicle-treated injured rats spent more time in the neutral zone than sham or EPO + MLT-treated rats. EPO treatment by itself did not improve disinhibition (two-way ANOVA with Bonferroni correction, *p < 0.05, **p < 0.01, ***p < 0.001).

We assessed open field behavior to quantify activity and disinhibition. Compared to sham controls (*n* = 29), rats subjected to prenatal injury (*n* = 23) were much more mobile, which was particularly evident in the last 5 min of the 15-min observation period (Figure 1A). Interestingly, compared to vehicle-treated rats with prenatal injury, EPO + MLT normalized the hypermobility (*n* = 28, two-way ANOVA, *p* = 0.022), whereas EPO alone (*n* = 15) did not. After prenatal injury, adult rats were also disinhibited. Specifically, those with *in utero* injury showed a lack of environmental awareness by spending more time immobile in the arena center compared to shams (*p* = 0.03; Figure 1B). Similarly, prenatal injury had a significant effect on disinhibition, with sham rats exploring the peripheral zone for significantly longer periods compared to vehicle-treated injury rats (*p* = 0.001; Figure 1C), and spending less time in the neutral zone (*p* = 0.041; Figure 1D). Treatment with EPO + MLT, but not EPO alone, normalized total time spent in the peripheral (*p* = 0.024) and neutral zones (*p* = 0.038; Figure 1), consistent with typical rat behavior, appropriate anxiety and general avoidance of open areas.

### EPO + MLT Normalizes Hindlimb Deficits After Prenatal Injury

**Figure 2:**
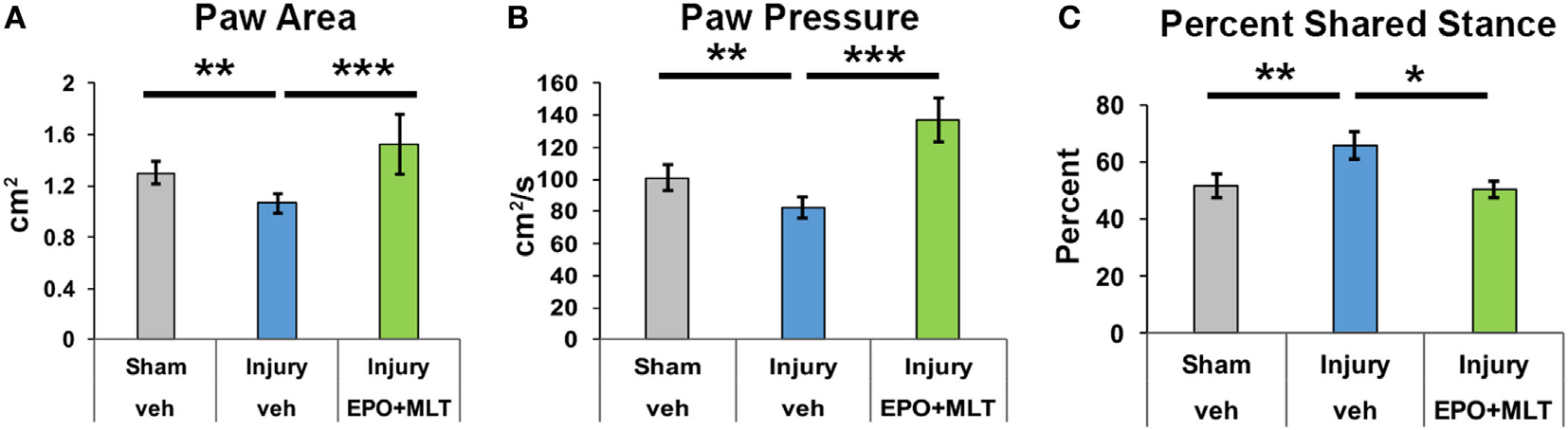
Prenatal injury impairs gait performance. **(A)** After prenatal injury, vehicle-treated rats contact their hindpaws with less area than sham or erythropoietin (EPO) + melatonin (MLT)-treated injured rats. **(B)** Similarly, vehicle-treated injured rats contact hindpaws with less pressure than shams or EPO + MLT-treated rats. **(C)** The percent of shared stance is elevated in vehicle-treated rats with prenatal injury compared to shams, and EPO + MLT treatment normalizes gait and posture (two-way ANOVA with Bonferroni correction, *p < 0.05, **p < 0.01, ***p < 0.001).

After observing that EPO + MLT normalized hyperlocomotion and disinhibition in adult rats following prenatal injury, we performed a detailed computerized digital analysis of gait to determine if EPO + MLT could improve motor performance. Compared to shams (*n* = 18), after prenatal injury vehicle-treated adult rats exhibit an abnormal gait, stance and paw placement, with decreased paw area contact (*n* = 21, *p* = 0.012) and decreased paw pressure (*p* = 0.031) suggestive of toe-walking, and reduced percent shared stance (*n* = 21, *p* = 0.006) (Figure 2) consistent with spastic gait patterns observed in ambulatory children with CP. Significantly, neonatal combination therapy with EPO + MLT (*n* = 7) reverses deficits in stance (*p* = 0.035) and paw placement (area and pressure both *p* < 0.001), consistent with an improved gait kinematic efficiency with combination treatment.

### EPO + MLT Attenuates Deficits in Social Interaction

**Figure 3:**
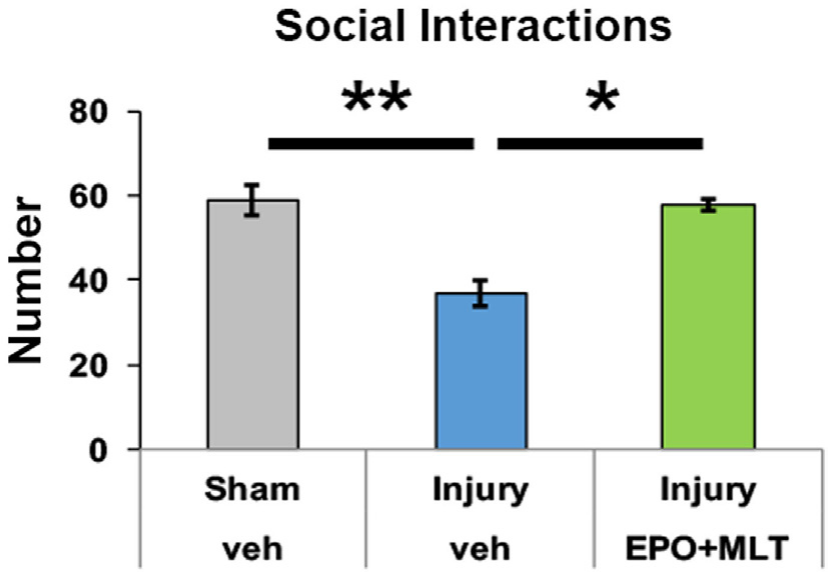
Prenatal injury impairs social interaction. After prenatal injury, pairs of vehicle-treated rats of the same sex but different litters have fewer social interactions than pairs of shams, or pairs of erythropoietin (EPO) + melatonin (MLT)-treated injured rats (two-way ANOVA with Bonferroni correction, *p < 0.05, **p < 0.01).

To quantify social interaction, pairs of sex, injury, and treatment-matched rats from different litters were observed and scored. The interrater reliability of social interaction scoring was 0.932. Sham (*n* = 18 rats in 9 pairs, *p* < 0.001) and injured rats treated with EPO + MLT (*n* = 8 rats in 4 pairs, *p* = 0.007) had significantly more social interactions during the observation period, including sniffing, playing, and grooming, compared to vehicle-treated rats with *in utero* injury (*n* = 14 rats in 7 pairs) (Figure 3). Significantly, EPO + MLT treatment ameliorated deficits in social drive and behavior.

### EPO + MLT Mitigates Deficits in Executive Function

To complement our assessment of gait, open field, and social behavior in adult rats with *in utero* injury, we completed a sophisticated assessment of visual discrimination and reversal learning in our animals to evaluate executive function. We began by validating the touchscreen platform in our model of *in utero* chorioamnionitis and assessed whether adult rats following prenatal insult could perform visual discrimination. Importantly, rats in all three treatment groups were successful in completing all aspects of touchscreen habituation and training.

**Figure 4:**
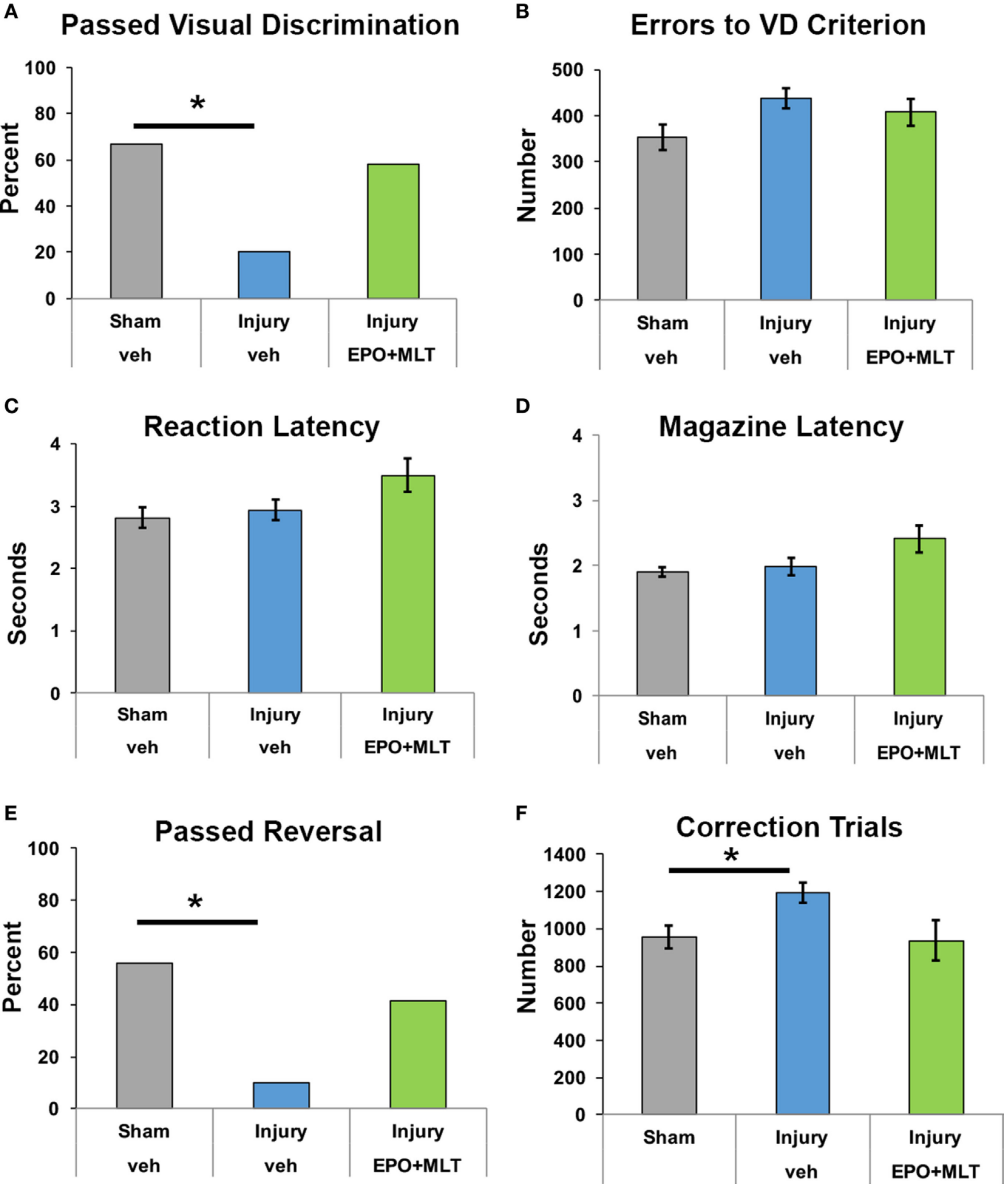
Touchscreen operant chamber testing reveals impaired cognition. **(A)** Fewer vehicle-treated rats after prenatal injury successfully reached passing criterion for visual discrimination, compared to shams or erythropoietin (EPO) + melatonin (MLT)-treated injured rats (*p < 0.05). **(B)** Shams, vehicle-treated rats with prenatal injury, and EPO + MLT-treated rats with prenatal injury all committed approximately the same number of errors during visual discrimination testing. **(C)** The reaction latency between the three groups did not differ. **(D)** Likewise, the magazine latency was similar for all three groups. **(E)** Significantly fewer vehicle-treated rats after prenatal injury were able to pass criterion for VD and reversal (*p < 0.05). **(F)** After prenatal injury, vehicle-treated rats required more correction trials in reversal learning paradigms than shams.

We first assessed cognitive performance on visual discrimination. Rats from each experimental group were able to perform VD, with 67% of sham (*n* = 27) and 20% of vehicle-treated injured rats (*n* = 20, *p* = 0.005) achieving passing criteria, compared to 58.3% of injured rats treated with EPO + MLT (*n* = 12, *p* = 0.11; Figure 4A). After assessing overall performance and pass rate, we then analyzed the number of errors throughout the visual discrimination paradigm as a more rigorous and granular metric of task performance. Notably, for those rats completing VD, a similar number of errors to achieve passing criteria was noted across injury and treatment groups (Figure 4B). As expected, all rats had comparable reaction time and magazine latency (Figures 4C,D) throughout the VD paradigm.

Upon successful completion of VD, rats were evaluated for reversal learning. Vehicle-treated injured rats were significantly impaired and fewer passed the overall learning paradigm compared to sham and EPO + MLT-treated rats. Specifically, only 10% of vehicle-treated injured animals successfully passed criteria for VD and reversal (*p* = 0.046) compared to 55.5% of sham and 41.5% of injured animals treated with EPO + MLT (*p* = 0.07; Figure 4E). Notably, injured animals treated with vehicle required more correction trials compared to shams (*p* = 0.034). Injured rats treated with EPO + MLT showed a trend toward fewer correction trials (*p* = 0.077), compared to vehicle-treated rats (Figure 4F). Further analyses of the maladaptive learning in the reversal paradigm consistent with a lack of cognitive flexibility, indicated a trend for improved performance during both perseveration and learning phases of the reversal paradigm. These results show that touchscreen testing can be used in PBI to distinguish complex behavior related to executive function and learning and that EPO + MLT can at least partially reverse the reduced cognition present after prenatal injury.

## Discussion

In this investigation, we tested the efficacy of combined EPO + MLT for neurorepair of the deficits associated with CP using translatable outcome measures, with the goal of facilitating rapid transition to neonatal clinical trials. To begin, we capitalized on a preclinical platform and model of CP that accurately recapitulates the multi-faceted pathophysiology of early CNS injury, including an intact maternal-placental-fetal axis, and sustained deficits in adult animals in multiple functional domains. These investigations reflect recent clinical epidemiological progress that indicates most CP arises from prenatal injury and that only 12% of cases arise from intrapartum insults (7). Consistent with clinical data, the preclinical findings reported here reaffirm the concept that chorioamnionitis concomitant with placental insufficiency results in dynamic, multifactorial, and permanent changes to the CNS culminating in functional deficits in mature rats. Indeed, this prenatal insult causes significant chronic behavioral, social, executive function, and gait deficits in adult rats, similar to those observed in children with CP (46, 62, 71). This model that incorporates intrauterine chorioamnionitis is one of very few preclinical models to induce persistent gait and neurocognitive deficits in the mature CNS (40, 70).

Mechanistically, compelling evidence suggests EPO and MLT have significant promise as potential synergistic interventions for neonates at high risk of CP. EPO and MLT have multiple over-lapping and complementary mechanisms of action. As developmentally regulated growth molecules, both EPO and MLT enhance neuronal and oligodendroglial survival and differentiation after CNS injury, suppress toxic cell death pathways, reduce free radicals, and mitigate inflammation from neonatal CNS infection (44–55). Unlike EPO, which is produced predominantly by the kidney and neural cells after birth (84), MLT is an endogenous indoleamine that is produced by the pineal gland postnatally and classically reported to regulate circadian rhythms. It is a direct antioxidant and free radical scavenger and also has indirect actions to increase the production of antioxidant enzymes including glutathione peroxidase (GP) and superoxide dismutase (SOD) (85). Notably, preterm infants with PBI, including those initiated *in utero* by choriomanionitis, are known to have reduced levels of GP and SOD in both the brain and lung (86).

The combination of EPO and MLT also has direct anti-inflammatory and immunomodulatory properties integral to their beneficial effects. Impaired regulation of immune responses is detrimental to multiple pregnancy outcomes, including preterm birth. It is plausible that both EPO and MLT link maternal, placental, and fetal physiological cell signaling through mechanisms of entrainment and direct biological actions (87). Interestingly, MLT is synthesized in higher concentrations within the placenta than the pineal gland (87, 88). Specifically, cyto- and syncytiotrophoblasts from the placenta contain two enzymes, serotonin *N*-acetyltransferase and *N*-acetylserotonin methyltransferase, which metabolize serotonin to MLT. Once in the circulation, MLT can increase phagocytosis, antigen presentation, and exert antioxidant effects (87, 89). Indeed, both EPO and MLT are known to affect Th1/Th2 ratio, Th17, neutrophils, and microglia, major cellular mediators of chorioamnionitis and PBI (87, 90). Through separate signal transduction, CNS inflammation actually reduces innate CNS EPO and MLT production, thereby diminishing endogenous neurorepair (91). In this context, similar to exogenous EPO therapy, exogenous MLT administration after birth may supplement innate levels by the pineal gland and replace a MLT deficit arising from premature separation from placental sources and/or induced inflammation from intrauterine infection or injury.

Erythropoietin and MLT also promote the genesis, survival, and differentiation of neural cells in the developing and mature CNS and reduce calpain-mediated injury. Previously, we have shown that sustained excess calpain activity is an important mechanism of injury in the immature CNS (45, 47, 48) and that extended EPO treatment mitigates calpain-mediated damage (48). Specifically, calpain degrades CNS molecules and proteins essential for the formation of cerebral circuits, including neurofilaments, myelin basic protein, and the potassium chloride cotransporter KCC2 (48). Therefore, through EPO and MLT together, it may be possible to cumulatively preserve more axon-myelin structural units, including those in major cerebral white matter and corticospinal tracts, by inhibiting detrimental protease expression, preserving structural connectivity, and restoring inhibitory neural networks. Indeed, it is through this action on structural and functional connectivity, neural conduction, and excitatory/inhibitory balance of fundamental circuitry that this combination of therapy likely improves motor and cognitive function into early adulthood (P90) following prenatal injury (46–48, 71).

Recently, it has been demonstrated that EPO has an additional novel mechanism in regulation of homeostatic plasticity and synaptic strength (92). Together, with previous reports on the modulation of inhibitory circuitry in brain regions key to higher order brain function and structural connectivity, this effect on synapses provides an additional novel molecular mechanism supporting the improvement in cognition and behavior shown here, and the normalization of the trajectory of brain development after perinatal injury (47, 66, 71, 72, 92–94). Similarly, complimenting the EPOR distribution on glia, and neurons, and the importance of receptor/ligand balance in the developing brain (55), MLT receptors, MT1 and MT2, are present in regions of the brain that are important to cognition and memory, including the hippocampus and frontal cortex and are similarly regulated by endogenous MLT (69, 87, 95, 96). In rodents, several studies have reported improved social behavior, anxiolytic, antidepressant, and memory-facilitating effects of exogenous MLT related to modulation of essential neurotransmitters and their receptors, including GABAergic, dopaminergic, glutamatergic, cholinergic, and noradrenergic transmission (69, 97–100). Consistent with our data, studies in mice confirm a MLT-induced decrease in open field hyperlocomotion (69). Indeed, our data also matches prior studies demonstrating that MLT administration in similar dosing ranges reverses ketamine-induced deficits in social interaction and memory impairment (69). Significantly, these investigators found that mitigation of social deficits only occurred with dose-dependent, repeated administration of MLT. Interestingly, in our studies, the combination of EPO + MLT was able to reverse abnormal open field behavior, whereas EPO alone was not. This highlights that, in specific microenvironments, a combination of agents may be more effective than one agent alone, and through repair of white matter, synapses, structural connectivity, neural network efficiency, and multiple neurotransmitter systems, EPO and MLT together may additively improve multiple pillars of cognition and behavior.

This is the first time to our knowledge that touchscreen platforms have been used with preclinical models of CP and PBI. Given that many children with CP have sensorimotor *and* cognitive deficits, there is a need to study strategies that address both functional domains. Touchscreen assessment is a highly translatable outcome measure also utilized in human trials and the Cambridge Neuropsychological Test Automated Battery is regularly used for neuropsychological testing of children and adults (76, 80). Our results demonstrate that EPO + MLT partially mitigates deficits of cognition, specifically executive function and reversal learning. These data support the clinical literature showing that very preterm children and adolescents are at high risk for executive function deficits that only become apparent with increasing cognitive demands. Specifically, compared to healthy born peers, preterm adolescents scored significantly lower in the most demanding levels of working memory, planning, cognitive flexibility, and verbal fluency tasks, despite no group differences being detected at lowest demand levels (101).

We also found *in utero* injury influenced exploratory behavior in mature animals, with EPO + MLT normalizing excess center mobility and resting time in an open field. These data are consistent with prior studies demonstrating improvements in exploratory behavior and resting time with MLT treatment, consistent with normalization of disinhibition, hyperlocomotion, and depression-like behavior (102). Indeed, prior reports suggest that MLT exerts a long-term effect on striatal dopamine content by enhancing monoamine synthesis (102, 103). While that investigation was performed in older rats, it is possible that normalized monoamine system development might improve motor coordination and cognition. Similarly, MLT also acts as a 5HT_2A_ antagonist in the hippocampus, and through the regulation of 5HT release may also impact complex behaviors related to anxiety, behavioral inhibition, and locomotion (102, 104). Notably, children with CP often exhibit spasticity and difficulty with selective motor control. Selective motor control is regulated predominantly by descending serotonin pathways that innervate the lumbar spinal cord on E18 in rats (105), the same age as the prenatal injury used here. Importantly, signaling *via* 5HT_2A_ upregulates spinal KCC2 levels (106). Serotonin signaling is also integral to cognitive flexibility (107). Thus, repairing myelination, axons, KCC2 levels and preserving homeostasis of serotonin signaling may be key mechanistic conversion points of EPO + MLT combination therapy and critical to minimizing spasticity, loss of selective motor control, and preserving cognition in children with CP from prematurity.

In conclusion, EPO + MLT are plausible targeted pharmacotherapies that specifically enhance neurorepair *via* novel and disease-specific molecular mechanisms. Receptor and non-receptor-mediated pathways underpin the multiple neuroprotective effects of MLT and EPO that include supporting mitochondrial function, and post-injury plasticity, and antioxidant, anti-apoptotic, and anti-inflammatory actions (108, 109). Together with normalization of social interaction, these data suggest a plausible treatment strategy to address multiple realms of cognition and behavior. In conjunction with data presented in this study, coupled with EPO and MLT’s known safety profile, multiple beneficial mechanisms of action, ability to penetrate the brain and organelles, and ease of administration, EPO and MLT in combination merits thoughtful consideration of clinical trials for preterm infants with brain injury.

## Ethics Statement

This study was carried out in accordance with the recommendations and approval of The Institutional Care and Use Committee (IACUC) at the University of New Mexico Health Sciences Center, Boston Children’s Hospital, and Johns Hopkins University.

## Author Contributions

Conception and design: SR and LJ. Acquisition of data: LJ, AO, FC, TY, JK, GF, and AW. Analysis and interpretation of data: LJ, SR, and FN. Drafting the article: SR and LJ. Critically revising the article: all authors. Reviewed submitted version of manuscript: all authors. Study supervision: SR and LJ. SR’s previous institution and location at the beginning of this study: Department of Neurosurgery, Boston Children’s Hospital, Harvard Medical School, Boston MA.

## Conflict of Interest Statement

The authors declare that the research was conducted in the absence of any commercial or financial relationships that could be construed as a potential conflict of interest.
